# Occurrence of *Aelurostrongylus abstrusus* and *Troglostrongylus brevior* in domestic cats in Greece

**DOI:** 10.1186/s13071-015-1200-z

**Published:** 2015-11-14

**Authors:** Anastasia Diakou, Angela Di Cesare, Luciano A. Barros, Simone Morelli, Lenaig Halos, Frederic Beugnet, Donato Traversa

**Affiliations:** Laboratory of Parasitology and Parasitic Diseases, School of Veterinary Medicine, Faculty of Health Sciences, Aristotle University of Thessaloniki, 54124 Thessaloniki, Greece; Faculty of Veterinary Medicine, Teaching Veterinary Hospital, Località Piano d’Accio snc, 64100 Teramo, Italy; Faculdade de Medicina Veterinaria MSV/UFF, Rua Vital Brasil Filho, 64 Icaraí, Niterói, Rio de Janeiro, 24230-340 Brazil; Merial S.A.S, 29 Av Tony Garnier, 69007 Lyon, France

**Keywords:** *Aelurostrongylus abstrusus*, *Troglostrongylus brevior*, Lungworms, Greece, Greek islands, Domestic cats

## Abstract

**Background:**

Despite the evidence that Mediterranean Europe offers suitable conditions for the biology of felid respiratory metastrongyloids, no updated data on the presence of felid lungworms are available for Greece. Although the cat lungworm *Aelurostrongylus abstrusus* is considered as enzootic in domestic cats (*Felis silvestris catus*) living in some areas of continental Greece, conversely, *Troglostrongylus brevior*, has only been reported in the island of Crete. The present study aimed to evaluate the occurrence of *Aelurostrongylus abstrusus* and *Troglostrongylus brevior* in domestic cats from four different Greek locations including islands where European wildcats (*Felis silvestris silvestris*), believed to be the natural reservoir of *T. brevior*, are considered absent.

**Methods:**

Faeces were collected from 125 stray cats in the city of Athens, and in Crete, Mykonos and Skopelos Islands, and examined by copromicroscopic techniques for the presence of lungworm larvae. When present, larvae were morphologically and molecularly identified.

**Results:**

The occurrence of *A. abstrusus* and *T. brevior* was confirmed in 10 (8 %) and 7 (5.6 %) of the samples, respectively. In particular, *T. brevior* was detected in domestic cats in the city of Athens, and in Mykonos and Skopelos Islands, where wildcats are not present.

**Conclusions:**

This information illustrates that *T. brevior* may infect domestic cats regardless of the presence of the natural host. Considering the relevant clinical impact of this nematode especially in young animals, it is advisable to include troglostrongylosis in the differential diagnosis of cat respiratory diseases also where this parasite is unexpected.

## Background

The cat lungworm *Aelurostrongylus abstrusus*, i.e. the most important respiratory parasite of domestic cats (*Felis silvestris catus*) worldwide, has been traditionally considered to be the only metastrongyloid affecting *F. s. catus* [1, 2]. Nonetheless, other respiratory nematodes have recently been recorded from *F. s. catus* either after a long time since the first description, or for the first time. Some of them, e.g. *Troglostrongylus subcrenatus* or *Oslerus rostratus*, still remain of minor and limited relevance under an epizootiological standpoint and are likely to be confined in their wild hosts [1–4]. Conversely, *Troglostrongylus brevior* has gained high attention in the past few years, for its clinical relevance, especially in young animals and for an apparent expansion in populations of domestic cats [1, 4, 5].

*Aelurostrongylus abstrusus* affects domestic cats worldwide, with high prevalence in certain areas [1]. In Greece, the parasite has been found in 1.6 % of the cats in the North of the country [6], and since then it has been sporadically detected in routine parasitological examination of cats (Diakou, unpublished data).

*Troglostrongylus brevior* was first described in the last century from two species of wild felids in Palestine [7]. It was then found in one European wildcat (*Felis silvestris silvestris*) from central Italy and in an animal that the author did not clarify whether it was a feral cat or a wildcat [8]. No other records of *T. brevior* were published in the peer-reviewed literature until 2010, when this lungworm was recorded for the first time in two domestic cats living in Ibiza Island (Spain) [9]. The report from Ibiza brought troglostrongylosis in domestic cats to the fore and since then, this infection has been increasingly recorded in other European islands, i.e. Sicily, Sardinia and Crete [5, 10, 11], and in areas of continental Italy [3, 4, 12–16].

**Fig. 1:**
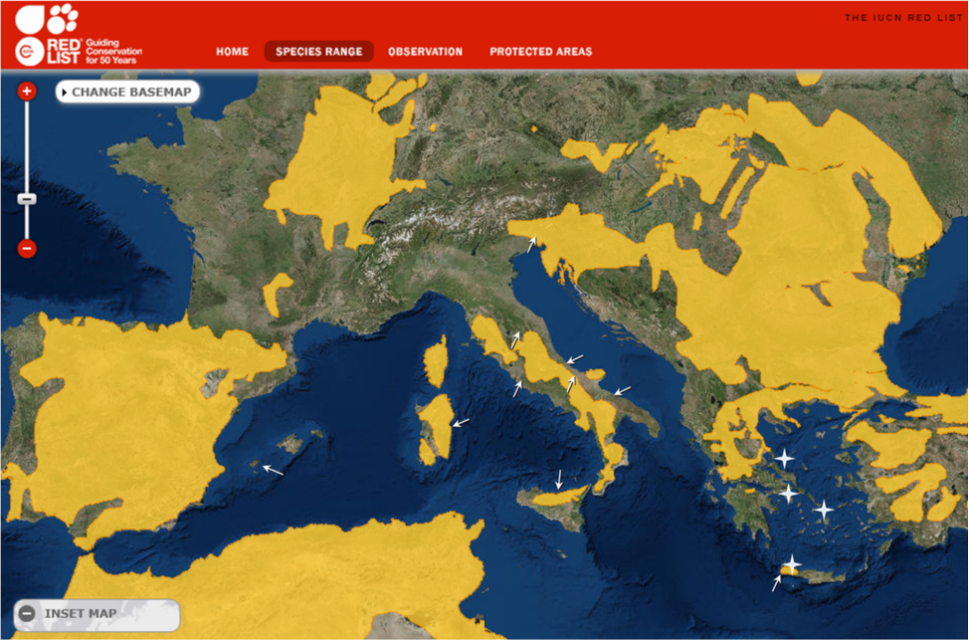
Map of the range of *Felis silvestris silvestris* in Southern and Central Europe (modified from the site of the International Union for Conservation of Nature (IUCN, www.iucnredlist.org) Arrows indicate the areas were *Troglostrongylus brevior* has been documented in domestic cats; asterisks indicate the study areas

In Italy and Greece, *T. brevior* until now has been described in domestic cats living in areas where the presence of wildcats is documented (Fig. 1) [3–5, 10–17]. A range of reasons indicate that wildcats may be the natural hosts of *T. brevior*, and that a spill-over of this parasite to domestic cats may occur in certain ecological niches [1, 2, 18].

Despite the evidence that Mediterranean Europe offers suitable conditions for the biology of felid respiratory metastrongyloids, no updated information is available for the occurrence of *A. abstrusus* in Greece, that has been thus far previously reported only in the northern territories [6]. Additionally, only one record exists for *T. brevior*, i.e. in one kitten from Crete Island [11].

The present article aims to update current knowledge on cat lungworms in Greece, describing the occurrence of *A. abstrusus* and *T. brevior* in domestic cats from continental and insular regions. Epizootiological and clinical implications of the findings are discussed in relation to different environmental habitats in Greece, both in areas of presence or absence of wildcats.

## Methods

### Study design and faeces collection

In June and July 2015 individual faecal samples of 125 stray cats were collected in four geographic locations in continental and insular Greece, i.e. the city of Athens (23 cats), and the islands of Crete (34 cats), Mykonos (43 cats) and Skopelos (25 cats). These locations were selected based on a study design (see Acknowledgments) including both continental and insular touristic areas of Greece, with varying environments, i.e. dry (Crete, Mykonos), forested (Skopelos) and highly urbanised (Athens) areas, and on an axel extending from the south to the north of the country.

Individual faeces were collected from each single cat after a rectal paediatric glycerine enema. All animals were examined with the consent of the local Municipality Authorities.

### Parasite detection and identification

The faecal samples were examined by classical copromicroscopic examination, i.e. ZnSO_4_ flotation and Baermann method [19, 20]. The larvae found at the faecal examinations were morphologically studied, measured and identified according to existing morphological descriptions and keys [1, 21, 22]. In addition, DNA was extracted from the purified larvae and molecular identification was conducted using a newly developed molecular assay for the simultaneous detection of *A. abstrusus* and *T. brevior* specific DNA [23]. In brief, a multiplex semi-nested PCR using a universal set of primers (i.e. NC1-NC2) in the first step and a combination of forward primers specific for cat respiratory nematodes along with NC2 primer was used [23].

## Results

**Fig. 2:**
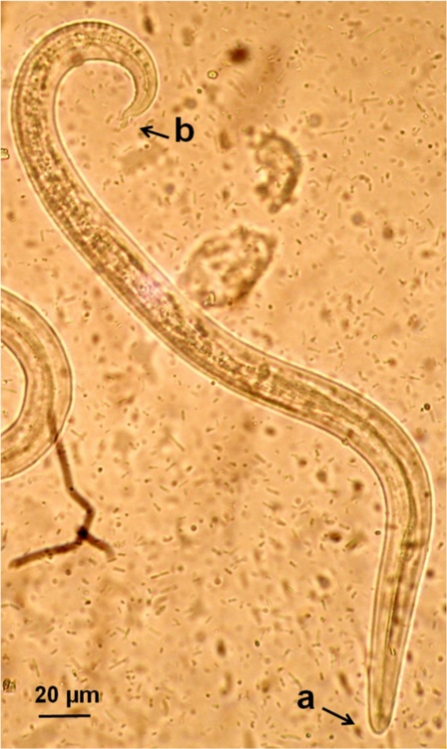
First stage larva (L1) of *Aelurostrongylus abstrusus* from a cat with aelurostrongylosis. Note the blunt anterior extremity with a terminal oral opening (**a**) and the deep dorsal incisure, ventral incisure and three knob-like appendages at the posterior extremity (**b**)

**Fig. 3:**
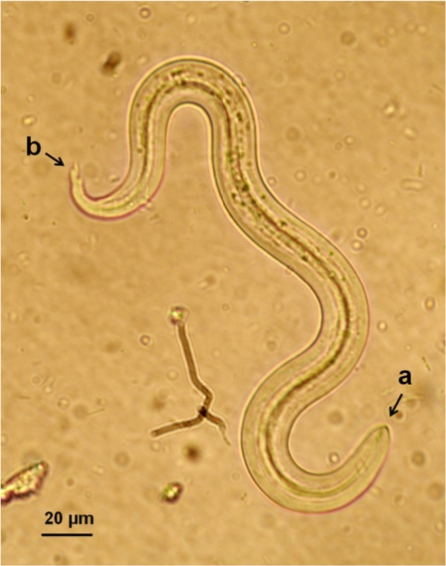
First stage larva (L1) of *Troglostrongylus brevior* from a cat with troglostrongylosis. Note the pointed anterior extremity with a subterminal oral opening (**a**) and the dorsal and ventral incisure at the posterior extremity (**b**)

**Fig. 4:**
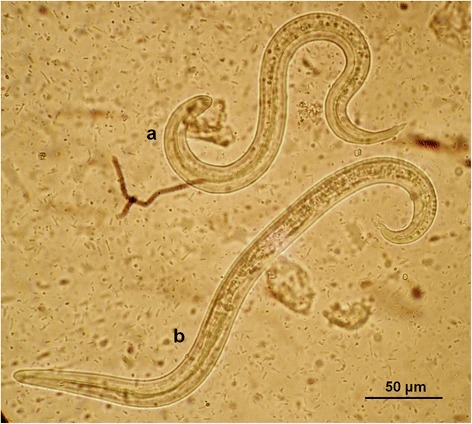
First stage larvae (L1) of *Troglostrongylus brevior* **a** and *Aelurostrongylus abstrusus* **b** from a cat with a mixed infection

At the copromicroscopic examination lungworm larvae were retrieved in the faeces of 15 cats (12 %) living in all examined sites, specifically in 5 (21.7 %), 1 (2.9 %), 7 (16.3 %) and 2 (8 %) of the cats from Athens, Crete, Mykonos and Skopelos, respectively (Table 1). The identification of the larvae in each positive sample was confirmed both by morphological examination and PCR. Larvae of *A. abstrusus* (Fig. 2) were found in a total of 10 (8 %) cats, i.e. 4 in Athens, 1 in Crete, 3 in Mykonos and 2 in Skopelos, while larvae of *T. brevior* (Fig. 3) were found in 7 (5.6 %) cats, i.e. in 1 in Athens, 5 in Mykonos and 1 in Skopelos. Of the 15 positive cats, 13 had a monospecific infection by either *A. abstrusus* (8 animals) or *T. brevior* (5 animals). Mixed infections (Fig. 4), were detected in two cats, one in Mykonos and one in Skopelos.

**Table 1:** Prevalence (% ± 95 % confidence interval, CI) of *Aelurostrongylus abstrusus* (Aab) and *Troglostrongylus brevior* (Tb) in cats examined from four different areas of Greece

Area	No. of cats	Aab (% ± CI)	Tb (% ± CI)	Total (% ± CI)
Athens	23	4 (17.4 ± 7.9)	1 (4.3 ± 4.2)	5 (21.7 ± 17)
Crete	34	1 (2.9 ± 2.8)	0	1 (2.9 % ± 5.6)
Mykonos	43	3 (7 ± 3.8)	5 (11.6 ± 4.8)	7^a^ (16.3 % ± 11)
Skopelos	25	2 (8 ± 5.4)	1 (4 ± 3.9)	2^a^ (8 % ± 10)
Total	125	10 (8 ± 2.4)	7 (5.6 ± 2)	15^a^ (12 % ± 2.9)

^a^Mixed infections by both parasites were recorded in 2 cats, 1 in Mykonos and 1 in Skopelos

## Discussion

The present data demonstrate that cats living in various Greek islands and in the continental territory of Athens may harbour *A. abstrusus* and *T. brevior*, in single or mixed infections, and with high infection rates in some areas (e.g. Athens, Mykonos). Most importantly, this report provides evidence that domestic cats may harbour *T. brevior* in regions where the natural host [24, 25], i.e. the wildcat, is absent. The high level of infection with lungworms in the examined stray cats shows the important role that free-roaming animals may have in the dissemination of these parasites. In fact, the level of veterinary care in stray cats is generally low, thus these animals remain untreated for a long time and disseminate their parasites in the environment. Hence, they act as a source of infection for the intermediate and paratenic hosts, increasing the risk of infection in other susceptible hosts living in the same regions.

The present study adds new knowledge on the geographical distribution of the cat lungworm *A. abstrusus* in Mediterranean Europe, as no updated and multi-site information were available for Greece and no data were obtained for the study areas thus far. It is here shown that cat aelurostrongylosis may occur with relatively high infection rates in some parts of Greece (Table 1), i.e. higher than the values previously recorded in the north of the country [6]. The only information available for *T. brevior* in Greece is a single case report from Crete [11]. This current study shows that this nematode can infect free-roaming domestic cats in continental Greece and islands other than Crete, in a range of latitudes extending from 35°51’N (Crete) to 39°12’N (Skopelos) and longitudes from 23°72’E (Athens) to 25°35’E (Mykonos). Interestingly, both *A. abstrusus* and *T. brevior* were found in varying environments, e.g. dry (Mykonos), forested (Skopelos) and highly urbanised (Athens) areas. It is important to note that the dry climate and poor vegetation characterising Mykonos and the urban environment of Athens do not represent an obstacle to the life-cycle of these mollusc-transmitted nematodes. On the contrary, the highest presence of both lungworms was recorded in these two study areas, thus indicating that the life-cycle of feline lungworms can be completed, as long as even limited habitats ensure the required conditions for the larval and intermediate host survival.

These results demonstrate that *T. brevior* is present in more geographical areas than previously known and that, in given regions, domestic cats harbour *T. brevior* regardless of the occurrence of wild reservoirs. At the moment it is still not ultimately established: (i) to what extent *T. brevior* occurs in domestic cats living in countries other than Italy, which is the region where this nematode has been recorded more frequently thus far [26]; (ii) if the reports of the last few years are due to a possible affiliation of *T. brevior* in domestic felids mostly in confined areas; and/or (iii) whether some epizootiological factors enforce infections of domestic cats with lungworms of wildlife.

The hypothesis that some drivers may have recently promoted an overflow of *T. brevior* from wildlife to domestic cats is realistic, based on different epizootiological, clinical and biological evidence [1, 4, 26]. As a key example, in a recent survey, only one cat of about 200 examined animals in northern Italy scored positive for *T. brevior*. This cat lived in the easternmost area of the only region of northern Italy where *F. s. silvestris* is present [4]. At the same time, cats examined in another northern territory of Italy where wildcats are absent, were highly infected with *A. abstrusus* but *T. brevior* was not found [4]. The same study showed that, in some mountainous and hilly areas of central Italy, aelurostrongylosis and troglostrongylosis have a similar distribution [4] despite the fact that *T. brevior* in domestic cats of the same regions was negligible until about ten years ago [3].

It is likely that under particular conditions (e.g. presence of infected wildcats facilitating the spread of infection, boundaries of islands or mountainous regions) *T. brevior* can change its usual affiliation and infect domestic cats, as in the case in Italy and Crete [1, 11, 26, 27]. It is difficult to draw a definitive explanation on the occurrence of cat troglostrongylosis in regions where the natural host responsible for spread of infection (i.e. *F. s. silvestris*) is not documented, i.e. Ibiza Island [9] and the Greek areas herein examined. The presence of wildcats in the Balearic Islands is uncertain and hypothesized only in the island of Mallorca [17, 28, 29]. Similarly, in Greece, populations of *F. s. silvestris* live only in forested areas and wetlands of continental regions [17], and in the Chania region of Crete Island (Fig. 1), i.e. where the first case of troglostrongylosis in a domestic cat from Greece has been recently described [11]. It is possible that, in given regions, this parasite has adapted to domestic cats in prehistorical times, before the present coastal arrangement appeared (c. 7000 BC - 4000 BC) [30], when the wildcat or a common ancestor was likely present. In such conditions, the nematode might have remained confined in domestic cats in geographical segregation, such as small islands (e.g. Ibiza, Mykonos), thus explaining why it has remained practically unknown until the recent descriptions. It is also possible that cats travelling with their owners in recent years from endemic areas like Italy, to popular and touristic spots could have imported this lungworm, or that movements of paratenic hosts (migratory birds, rodents moved with international trade of goods in ships, etc.) may have introduced infective stages of *T. brevior*. The absence of *T. brevior* in cats examined in Crete, despite the fact that this parasite is present on the island [11], could be due to a range of reasons. Indeed, *T. brevior* is in general less common than *A. abstrusus*, thus its absence in 34 examined cats is not surprising. The number of sampled animals could have reduced the chance of detection if this nematode has a limited distribution in local domestic cats and is still confined in wildlife. Moreover, most examined cats were from a different area (Stavros) from where the first case of troglostrongylosis was found in 2014 (city of Chania). It is important to note that *A. abstrusus* has been found here in only one cat, thus suggesting that lungworms in Crete may have a limited distribution compared to other areas.

Further studies could assist in exploring the genetic structure of *T. brevior* populations affecting both wild and domestic hosts from different geographic areas. These investigations could be important to understand if certain subpopulations of lungworms can selectively infect each different felid species in all enzootic areas or in given regions, or to the contrary, *F. s. catus* and *F. s. silvestris* share the same genetic populations of lungworms in all these areas. In fact, subpopulations of other strongylid species, i.e. hookworms and cyathostomins, were previously indicated to selectively infect different animal species [31, 32].

Finally, as a practical output, it is advisable that troglostrongylosis is definitively included in the differential diagnosis of respiratory diseases of cats, also in regions where *T. brevior* is unexpected. In fact, in the near future we could be faced with the spread of *T. brevior* in domestic cats, both where the natural hosts are expanding, i.e. central Italy [3, 4, 26, 27], and where wildcats are absent. This is of pivotal importance because a timely detection of *T. brevior* implies a prompt anthelmintic treatment with spot-on molecules that recently proved efficacious in treating cat troglostrongylosis in natural and/or experimental conditions, e.g. emodepside [14], eprinomectin [33, 34] and moxidectin [35].

## References

[1] Traversa D, Di Cesare A (2013). Feline lungworms: what a dilemma. Trends Parasitol.

[2] Traversa D, Di Cesare A. Cardio-pulmonary parasitic nematodes affecting cats in Europe: unraveling the past, depicting the present, and predicting the future. Frontiers Vet Sci. 2014; doi: 10.3389/fvets.2014.00011 10.3389/fvets.2014.00011 PMC4668853 26664917

[3] Di Cesare A, Di Francesco G, Frangipane Di Regalbono A, Eleni C, De Liberato C, Marruchella G (2015). Retrospective study on the occurrence of the feline lungworms *Aelurostrongylus abstrusus* and *Troglostrongylus* spp. in endemic areas of Italy. Vet J.

[4] Di Cesare A, Veronesi F, Grillotti E, Manzocchi S:, Perrucci S, Beraldo P (2015). Respiratory nematodes in cat populations of Italy. Parasitol Res.

[5] Brianti E, Gaglio G, Giannetto S, Annoscia G, Latrofa MS, Dantas-Torres F (2012). *Troglostrongylus brevior* and *Troglostrongylus subcrenatus* (Strongylida: Crenosomatidae) as agents of broncho-pulmonary infestation in domestic cats. Parasit Vectors.

[6] Haralabidis TS. Contribution in the study of cat parasites and their significance in Public Health. PhD Thesis. Scientific Yearbook of The Faculty of Veterinary Medicine, Aristotle University of Thessaloniki, Greece 1978;18: 237–379 (In Greek with English abstract)

[7] Gerichter CB (1949). Studies on the nematodes parasitic in the lungs of Felidae in Palestine. Parasitology.

[8] Paggi L (1959). Segnalazione, in Italia Centrale, di *Troglostrongylus* sp. parassita dei polmoni di felidi. Parassitologia.

[9] Jefferies R, Vrhovec MG, Wallner N, Catalan DR (2010). *Aelurostrongylus abstrusus* and *Troglostrongylus* sp. (Nematoda: Metastrongyloidea) infections in cats inhabiting Ibiza, Spain. Vet Parasitol.

[10] Tamponi C, Varcasia A, Brianti E, Pipia AP, Frau V, Pinna Parpaglia ML (2014). New insights on metastrongyloid lungworms infecting cats of Sardinia. Italy Vet Parasitol.

[11] Diakou A, Di Cesare A, Aeriniotaki T, Traversa D (2014). First report of *Troglostrongylus brevior* in a kitten in Greece. Parasitol Res.

[12] Brianti E, Gaglio G, Napoli E, Falsone L, Giannetto S, Latrofa MS (2013). Evidence for direct transmission of the cat lungworm *Troglostrongylus brevior* (Strongylida: Crenosomatidae). Parasitology.

[13] Di Cesare A, Frangipane Di Regalbono A, Tessarin C, Seghetti M, Iorio R, Simonato G (2014). Mixed infection by *Aelurostrongylus abstrusus* and *Troglostrongylus brevior* in kittens from the same litter in Italy. Parasitol Res.

[14] Di Cesare A, Iorio R, Crisi P, Paoletti B, Di Costanzo R, Dimitri CF (2015). Treatment of *Troglostrongylus brevior* (Metastrongyloidea, Crenosomatidae) in mixed lungworm infections using spot-on emodepside. J Feline Med Surg.

[15] Traversa D, Romanucci M, Di Cesare A, Malatesta D, Cassini R, Iorio R (2014). Gross and histopathological changes associated with *Aelurostrongylus abstrusus* and *Troglostrongylus brevior* in a kitten. Vet Parasitol.

[16] Traversa D, Lepri E, Veronesi F, Paoletti B, Simonato G, Diaferia M (2015). Metastrongyloid infection by *Aelurostrongylus abstrusus*, *Troglostrongylus brevior* and *Angiostrongylus chabaudi* in a domestic cat. Int J Parasitol.

[17] Yamaguchi N, Kitchener A, Driscoll C, Nussberger B. *Felis silvestris*. The IUCN Red List of Threatened Species. Version 2015.2. <www.iucnredlist.org>. Accessed 25 August 2015.

[18] Traversa D. Response to Otranto et al.: Lungworms in domestic and wild felids: dilemmas still persisting. Trends Parasitol. 2014;30:53-4 10.1016/j.pt.2013.10.008 24239265

[19] MAAF (1986). Manual of Veterinary Parasitological Laboratory Techniques, Ministry of Agriculture, Fisheries and Food (MAFF).

[20] Thienpont D, Rochette F, Vanparijs OFJ (1986). Diagnosing helminthiasis by coprological examination.

[21] Sloss MW, Kemp RL, Zajac AM (1994). Faecal examination: dogs and cats. Veterinary Clinical Parasitology.

[22] Brianti E, Giannetto S, Dantas-Torres F, Otranto D (2014). Lungworms of the genus *Troglostrongylus* (Strongylida: Crenosomatidae): neglected parasites for domestic cats. Vet Parasitol.

[23] Di Cesare A, Veronesi F, Frangipane Di Regalbono A, Iorio R, Traversa D (2015). Novel molecular assay for the simultaneous identification of neglected lungworms and heartworms affecting cats. J Clin Microbiol.

[24] Falsone L, Brianti E, Gaglio G, Napoli E, Anile S, Mallia E (2014). The European wildcats (*Felis silvestris silvestris*) as reservoir hosts of *Troglostrongylus brevior* (Strongylida: Crenosomatidae) lungworms. Vet Parasitol.

[25] Otranto D, Cantacessi C, Dantas-Torres F, Brianti E, Pfeffer M, Genchi C, Guberti V, Capelli G, Deplazes P. The role of wild canids and felids in spreading parasites to dogs and cats in Europe. Part II: Helminths and arthropods. Vet Parasitol. 2015; doi: 10.1016/j.vetpar.2015.04.020 10.1016/j.vetpar.2015.04.020 26049678

[26] Di Cesare A, Veronesi F, Traversa D. Felid lungworms and heartworms in Italy: more questions than answers? Trends Parasitol. 2015 in press. 10.1016/j.pt.2015.07.001 26507151

[27] Veronesi F., Traversa D., Lepri E., Morganti G., Vercillo F., Grelli D., Cassini R., Marangi M., Iorio R., Ragni B., Di Cesare A. Occurrence of lungworms in European wildcats (*Felis silvestris silvestris*) of central Italy. JWD. 2015 in press. 10.7589/2015-07-187 26967134

[28] Groves CP, Clutton-Brock J (2015). Feral mammals of the Mediterranean islands: documents of early domestication. The Walking Larder: Patterns of Domestication, Pastoralism, and Predation.

[29] Garcia-Perea R, Palomo LJ, Gisbert J, Blanco JC (2007). Felis silvestris Schreber, 1777. Atlas y Libro Rojo de los Mamíferos Terrestres de España.

[30] van Andel TH, Shackleton JC (1982). Late Paleolithic and Mesolithic Coastlines of Greece and the Aegean. J Field Archaeol.

[31] Hu M, Chilton NB, Zhu X, Gasser RB (2002). Single-strand conformation polymorphism-based analysis of mitochondrial cytochrome c oxidase subunit 1 reveals significant substructuring in hookworm populations. Electrophoresis.

[32] Traversa D, Kuzmina T, Kharchenko VA, Iorio R, Klei TR, Otranto D (2008). Haplotypic variability within the mitochondrial gene encoding for the cytochrome c oxidase 1 (cox1) of *Cylicocyclus nassatus* (Nematoda, Strongylida): evidence for an affiliation between parasitic populations and domestic and wild equid hosts. Vet Parasitol.

[33] Giannelli A, Brianti E, Varcasia A, Colella V, Tamponi C, Di Paola G (2015). Efficacy of Broadline® spot-on against *Aelurostrongylus abstrusus* and *Troglostrongylus brevior* lungworms in naturally infected cats from Italy. Vet Parasitol.

[34] Knaus M, Visser M, Chester TS, Rehbein S. Efficacy of Broadline® (Merial) against induced infection with *Troglostrongylus brevior* (Nematoda, Metastrongyloidea). Merial Abstract and Poster Book “New trends in the control of parasites in dogs and cats”, WAAVP, Liverpool, 16–20 August 2015, p. 87–89.

[35] Crisi PE, Traversa D, Di Cesare A, Luciani A, Civitella C, Santori D, Boari A. Irreversible pulmonary hypertension associated with *Troglostrongylus brevior* infection in a kitten. Res Vet Sci, in press, 2015; doi: 10.1016/j.rvsc.2015.08.019. 10.1016/j.rvsc.2015.08.019 26412548

